# Mesothelin-based CAR-T cells exhibit potent antitumor activity against ovarian cancer

**DOI:** 10.1186/s12967-024-05174-y

**Published:** 2024-04-18

**Authors:** Jing Guo, Xiaozhu Zeng, Yongjie Zhu, Dong Yang, Xudong Zhao

**Affiliations:** Department of Targeting Therapy & Immunology and Laboratory of Animal Tumor Models, Cancer Center and State Key Laboratory of Respiratory Health and Multimorbidity and Frontiers Science Center for Disease-Related Molecular Network, West China Hospital, Sichuan University, Chengdu, Sichuan China

**Keywords:** MUC16 (CA125), Mesothelin, CAR-T cells, Ovarian cancer

## Abstract

**Background:**

Ovarian cancer (OC) is characterized by its rapid growth and spread which, accompanied by a low 5-year survival rate, necessitates the development of improved treatments. In ovarian cancer, the selective overexpression of Mucin-16 (MUC16, CA125) in tumor cells highlights its potential as a promising target for developing anti-tumor therapies. However, the potential effectiveness of CAR-T cell therapy that targets MUC16 in ovarian cancer cells is unknown.

**Methods:**

The expression of MUC16 in viable OC cells was detected using immunofluorescence and flow cytometry techniques. A MSLN-CAR construct, comprising the MUC16-binding polypeptide region of mesothelin (MSLN), a CD8 hinge spacer and transmembrane domain, 4-1BB, and CD3ζ endo-domains; was synthesized and introduced into T cells using lentiviral particles. The cytotoxicity of the resultant CAR-T cells was evaluated in vitro using luciferase assays. Cytokine release by CAR-T cells was measured using enzyme-linked immunosorbent assays. The anti-tumor efficacy of the CAR-T cells was subsequently assessed in mice through both systemic and local administration protocols.

**Results:**

MSLN-CAR T cells exhibited potent cytotoxicity towards OVCAR3 cells and their stem-like cells that express high levels of MUC16. Also, MSLN-CAR T cells were inefficient at killing SKOV3 cells that express low levels of MUC16, but were potently cytotoxic to such cells overexpressing MUC16. Moreover, MSLN-CAR T cells delivered via tail vein or peritoneal injection could shrink OVCAR3 xenograft tumors in vivo, with sustained remission observed following peritoneal delivery of MSLN-CAR T cells.

**Conclusions:**

Collectively, these results suggested that MSLN-CAR T cells could potently eliminate MUC16- positive ovarian cancer tumor cells both in vitro and in vivo, thereby providing a promising therapeutic intervention for MUC16-positive patients.

**Supplementary Information:**

The online version contains supplementary material available at 10.1186/s12967-024-05174-y.

## Introduction

Mucin16 (MUC16), also known as carbohydrate antigen 125 (CA125), exhibits abnormally elevated expression in certain tumors such as ovarian cancer, breast cancer, pancreatic cancer and lung cancer [1–4]. Its elevated expression level is associated with cancer progression, metastasis, and poor prognosis for patients [5, 6]. Previous studies have reported that the expression of MUC16 is in normal cells is typically restricted by epithelial cell polarity [7, 8]. However, during the process of carcinogenesis, when cell polarity is dysregulated, MUC16 is expressed on the cell surface and interacts with various growth factors, and this feature of MUC16 is highly relevant to cancer [5, 9, 10]. As tumors form, the glycosylphosphatidylinositol-anchored cell-surface protein known as mesothelin binds MUC16 at *N*-glycosylation sites to promote cancer cell adhesion, leading to metastatic activation of tumors, including mesothelioma and ovarian cancer, in the pleura and peritoneum [11–13]. Given the importance of their interaction for the tumorigenicity of ovarian cancer cells, MUC16 and mesothelin have emerged as putative targets for developing treatments for peritoneal metastasis in epithelial ovarian cancer [14, 15].

Cancer treatments for MUC16 cancers are currently at various stages of progress. For example, clinical trials for the targeted monoclonal antibody drug Oregovomab and CAR T therapy (JCAR-020) for MUC16 are in progress [16, 17]. Furthermore, there are several drugs in development that target MUC16, such as bispecific T-cell engagers (BiTE) and antibody–drug conjugates (ADC) [9, 18]. However, no MUC16-targeted drugs have so far been approved by the FDA, due to their limited efficacy and low safety profile as treatments. Such antibody treatments target the circulating extracellular domain of MUC16 and may deplete the pool of circulating antibodies available for targeting cancer cells [5, 19].

Developments in CAR T cell therapy in the last 10 years have significantly enhanced its potential as a viable approach to develop effective treatments for many cancers. In earlier versions of such targeted drugs, the antigen recognition region in structures such as chimeric antigen receptors (CARs) have typically been composed of single-chain antibody fragments (scFv), predominantly sourced from murine sequences [20, 21], which could have consequences in the context of its use when treating humans. In addition to their inherent immunogenicity, scFv can also cause internalization and degradation of tumor antigens during the treatment process, leading to treatment failure [22, 23]. More recently, several studies have reported that the use of strategies exploiting endogenous ligand-receptor interactions in the construction of CAR T therapies can be highly effective to facilitate their ability to distinguish malignant tumor cells from non-cancer cells that weakly express or almost do not express the targeted antigen [24, 25]. In a recent study, CAR T cells were constructed using the N-terminal sequence of mesothelin. However, in vivo experiments utilizing mesothelin-based CAR T cells alone did not demonstrate effective cytotoxicity towards tumors in mice [26], however the underlying reasons were not clear.

Here, we have developed CAR-T cells that exploit the binding capacity of mesothelin to MUC16. We find that MSLN-CAR T cells effectively eliminate ovarian cancer tumor cells and stem-like cells in vitro, and this cytotoxic function is dependent on high expression levels of MUC16 on the tumor cell membrane. Furthermore, we found that MSLN-CAR T cells were effective to shrink tumors from ovarian cancer cell in immunocompromised mice. Our results suggest that ligand-receptor-based CAR T cell therapy that is designed to exploit the interaction between mesothelin and MUC16 may be effective as an ovarian cancer treatment.

## Materials and methods

### Cell culture

Human ovarian cancer cell lines OVCAR3 and SKOV3 were purchased from the National Infrastructure of Cell Line Resource (Kunming, China), while HEK-293T cells were sourced within our existing laboratory stocks. SKOV3 cells were cultured in McCoy 5A culture medium (Sigma-Aldrich, USA) supplemented with 10% FBS (Gibco, USA), while OVCAR3 medium was RPMI-1640 (Gibco, USA) supplemented with 0.01 mg/ml bovine insulin and 20% FBS, as recommended by the ATCC. HEK-293T cells and human immortalized umbilical endothelial cells (EAhy926) were cultured in Dulbecco’s Modified Eagle’s Medium (DMEM; Gibco, USA) with 10% FBS. Luciferase-expressing cell lines were derived through infection with pTomo-CMV-luciferase-IRES-puro lentivirus and subsequent selection with puromycin (1ug/ml, Gibco, USA) for 2 weeks. OVCAR3-luc and SKOV3-luc cells were cultured in serum-free stem cell medium consisting of DMEM/F12 (Gibco, USA) supplemented with EGF (20 ng/ml, Life Technologies, USA), bFGF (20 ng/ml, Life Technologies, USA) and B27(1×, Life Technologies, USA) to induce stem-like cancer cells (CSC), namely OVCAR3-CSC and SKOV3-CSC, respectively. All cells were cultured at 37 °C in a humidified incubator with 5% CO_2_ and routinely tested by PCR to be *Mycoplasma*-free. All cell lines were validated using Short Tandem Repeat (STR) profiling. Detailed STR information of the cell lines was presented in Additional file 6: Table S1.

### Lentivirus-mediated CAR construction and production

The coding sequences for MSLN (aa 290–362) polypeptide were synthesized (Tsingke Biotechnology Co., Ltd.) and fused to a CAR backbone comprising a human CD8 hinge spacer and transmembrane domain, 4-1BB costimulatory domain, CD3ζ and fused mKate2 via T2A sequence. Previous research has confirmed that the average dissociation constant (KD) of the MSLN fragment Fc fusion protein binding to MUC16 (CA125) in this study is approximately 3 nM, which is comparable to the affinity of natural mesothelin binding to MUC16 on OVCAR-3 cells [13]. The sequence comprising the entire CAR expression molecule was then cloned into the lentiviral vector pTomo (Addgene, #26291) and expressed under the control of a CMV promoter. As a negative control, a CD19-specific scfv (FMC63) sequence was constructed into the CAR backbone construct for experiments conducted in parallel that account for non-specific effects. For all constructs, lentiviral supernatant production was performed according to previous published reports [27, 28].

### CAR T cell production

Human peripheral blood mononuclear cells (PBMC) were collected from healthy donors with informed consent and isolated using RosetteSep™ Human T Cell Enrichment Cocktail (STEMCELL, Canada) according to the manufacturer’s protocol. T cells were cultured in advanced 1640 media (Gibco, USA) supplemented with 1× Glutamax (Gibco, USA), 10% FBS, 1× Penicillin–Streptomycin (Life technology, USA) and IL-2 (200 U/ml, PeproTech, USA); and stimulated with human CD3/CD28 Dynabeads (Life Technologies, USA) following the manufacturer’s instructions. To generate CAR T cells, T cells were cultured for 72 h and infected by lentiviral particles in the presence of LentiBOOST (Sirion Biotech, Germany). CAR expression, measured as the relative mKate2 signal ratio, was subsequently assessed 72 h later by flow cytometry to inform on dosage for subsequent experiments. The study was approved by the Biomedical Ethics Committee of West China Hospital of Sichuan University (Ethics Number: 2022151).

### Cytotoxicity assay and cytokine release assay

The cytotoxicity of CAR T cells was tested using a Luciferase Assay System (Promega, USA) at variable effector-to-target (E:T) ratios of 0.5:1, 1:1, 2:1 and 4:1. Briefly, 2 × 10^3^ target cells per well were seeded in 96-well plates with 100 μL medium, together with an equal volume of effector cells or control medium added according to the prescribed E:T ratios. For tests with CSCs, prior to cytotoxicity assays, such cells were adhered to plates overnight using laminin (Gibco, USA). After 20 h of co-culture, the supernatant was collected and used to determine the concentrations of IFN-γ (Invitrogen, KHC4021) and TNF-α (Proteintech, KE00154). The target cells were then lysed to test the cytotoxicity of CAR T cells according to the instructions of the manufacturer (Promega).

### RNA isolation and real-time PCR

Total RNA was extracted as described previously [29]. qPCR assays were performed with SYBR Selected Master Mix (Thermo Fisher, USA). The comparative cycle time (Ct) method was used to determine differences between samples, and the expression of target genes were normalized to 18S rRNA (2^−△△Ct^). The primer sequences are listed in Additional file 7: Table S2.

### Vector copy number

Vector copy number (VCN) per cell was determined by quantitative polymerase chain reaction (qPCR). qPCR assays were performed with SYBR Selected Master Mix (Termo Fisher, USA). Genomic DNA was extracted from 1 × 10^6^ MSLN-CAR T cells using isopropanol sedimentation and 100 ng DNA was used per qPCR reaction. qPCR amplification conditions and VCN analysis were performed as previously described [30, 31]. The primer sequences are listed in Additional file 7: Table S2.

### Immunofluorescence assays

To visualize membrane-bound MUC16 by immunofluorescence staining (IF), cancer cells (5 × 10^4^) were plated on coverslips in 24-well plates. For CSCs, prior to IF staining experiments, such cells were adhered to plates overnight using laminin (Gibco, USA), following which cells were subsequently incubated with anti-MUC16 antibody (sc-365002, Santa Cruz Biotechnology, USA) for one hour followed by washes and fixation with 4% PFA for 5 min. Thereafter, cells were washed three times and then incubated with goat anti-mouse secondary antibody (A-10677, Invitrogen, USA) for one hour at room temperature. Nuclei were stained with DAPI (1 μg/ml, Sigma) for 10 min. Fluorescent images were captured using a confocal microscope (Nikon, Japan).

### Flow cytometry

One million CAR T cells were stained with primary antibodies for antigen–antibody binding reaction. After 30 min of incubation in the dark, unbound antibodies and solutes were washed with a PBS rinse. This method was used to detect T cell transfection rates, with the following primary antibodies used: CD4 antibody (BioLegend Cat# 357408, USA), CD8 antibody (BioLegend Cat# 344714), CD45RO antibody (BioLegend Cat# 304206), CCR7 antibody (BioLegend Cat# 353214). Target cells were harvested and resuspended in cold PBS containing 2% FBS. The anti-MUC16 antibody was added to the cell suspension for incubation for 1 h. The cells were centrifuged, resuspended, and incubated with Cy3-labeled secondary antibodies in PBS containing 2% FBS for 30 min prior to two washes with PBS. For all flow cytometry experiments, appropriate IgG isotype controls were used to assess nonspecific staining. The specificity of MUC16 antibodies was confirmed by utilizing control IgG matched to the species. All flow cytometry data were collected using a BD LSRFortessa system (BD Biosciences, USA) and analyzed with FlowJo software (FlowJo, Oregon, USA). All flow antibodies are listed in Additional file 8: Table S3.

### Western blotting

Harvested cells were lysed in RIPA buffer, and protein concentrations were quantified using BCA protein assay kits (Beyotime, China). Total protein lysates from each sample were separated by 10% sodium dodecyl sulfate polyacrylamide gel electrophoresis and transferred to PVDF membranes (Millipore, USA). The membranes were blocked in Tris-buffered saline with 5% non-fat milk and then incubated 4 °C overnight with anti-MUC16 (sc-365002, Santa Cruz Biotechnology, USA), anti-CD133 (sc-365537, Santa Cruz), anti-CD44 (15675-1-AP, Proteintech, USA), and anti-Tubulin (MA5-16308, Thermo Fisher Scientific, USA) antibodies diluted in 5% bovine serum albumin (BSA). All horseradish peroxidase (HRP)-conjugated secondary antibodies were purchased from Sigma (USA) and used at a working dilution of 1:5000 in working solution. Blots were visualized with chemiluminescent HRP substrate (Millipore, USA). All the antibodies are listed in Additional file 8: Table S3.

### In vivo NCG mouse tumor models

In all in vivo studies, 6- to 8-week-old female NOD/ShiLtJGpt-*Prkdc*^*em26Cd52*^*Il2rg*^*em26Cd22*^/Gpt (NCG) mice were used. These were purchased from GemPharmatech Co., Ltd (Jiangsu, China). A total of 5 × 10^5^ OVCAR3-luc cells were injected intraperitoneally into NCG mice to establish xenograft models. Mice were intraperitoneally injected with 150 mg/kg d-Luciferin and Potassium Salt (BioVison) 10 min before they were placed in a chamber supplied with an 3% isoflurane gas mixture with oxygen (RWD, Shenzhen, China) for 2 min. The tumor burden was then measured by IVIS Spectrum (Lumina Xr, PerkinElmer, USA) and analyzed by Living Image software (Caliper Life Science). Subsequently, 5 × 10^6^ CD19-CAR T cells or MSLN-CAR T cells, at indicated doses, were injected either intraperitoneally or via tail vein injection on day 5 following tumor cell injection. The survival time of each mouse was recorded and the survival curve plotted using GraphPad Prism 8.3.0 software. Animal cohort sizes were determined on the basis of similar previous studies [32–34]. All animal studies were approved by the animal ethics committees of the West China Hospital of Sichuan University (20211458A).

### Immunohistochemical analysis and hematoxylin and eosin staining

Tissues were fixed with 4% paraformaldehyde, dehydrated with gradient ethanol baths, and finally embedded in paraffin. Sections of preserved tissue within paraffin blocks were then cut and stained with hematoxylin (0.2%) and eosin (1%) to assess histopathological changes in major organs after T cell infusion. For immunofluorescence staining experiments, tissue sections were first dewaxed and dehydrated, boiled in citrate buffer (pH 6.0) as an antigen retrieval step and then blocked using 10% normal goat serum at 37 °C for 1 h. Next, slides containing sections were incubated at 4 °C overnight with a solution of anti-CD3 antibody (ET1607-29, HUABIO, China), after brief washes, the sections were then incubated for 1 h with HRP-conjugated secondary antibodies (A6154-1ML, Sigma, USA) and with TSA Plus Fluorescein Reagent (1:50) for 10 min. Finally, nuclei were visualized by DAPI staining. Fluorescent images were taken using a confocal microscope (Nikon, Tokyo, Japan).

### Statistics

Data analysis and statistics were performed using GraphPad Prism software version 8.0 (Graphpad). Statistical comparisons were determined using Student’s *t* test (two groups) or one-way analysis of variance (ANOVA) (three groups or more) with Bonferroni’s multiple comparison test. Statistical significance in Kaplan–Meier survival curves was assessed with the Mantel-Cox log rank test. The levels of statistical significance were assessed at not significant (ns), P < 0.05 (*), P < 0.01 (**), P < 0.001 (***).

## Results

### MSLN-CAR T cells efficiently target ovarian cancer cells that overexpression MUC16

**Fig. 1:**
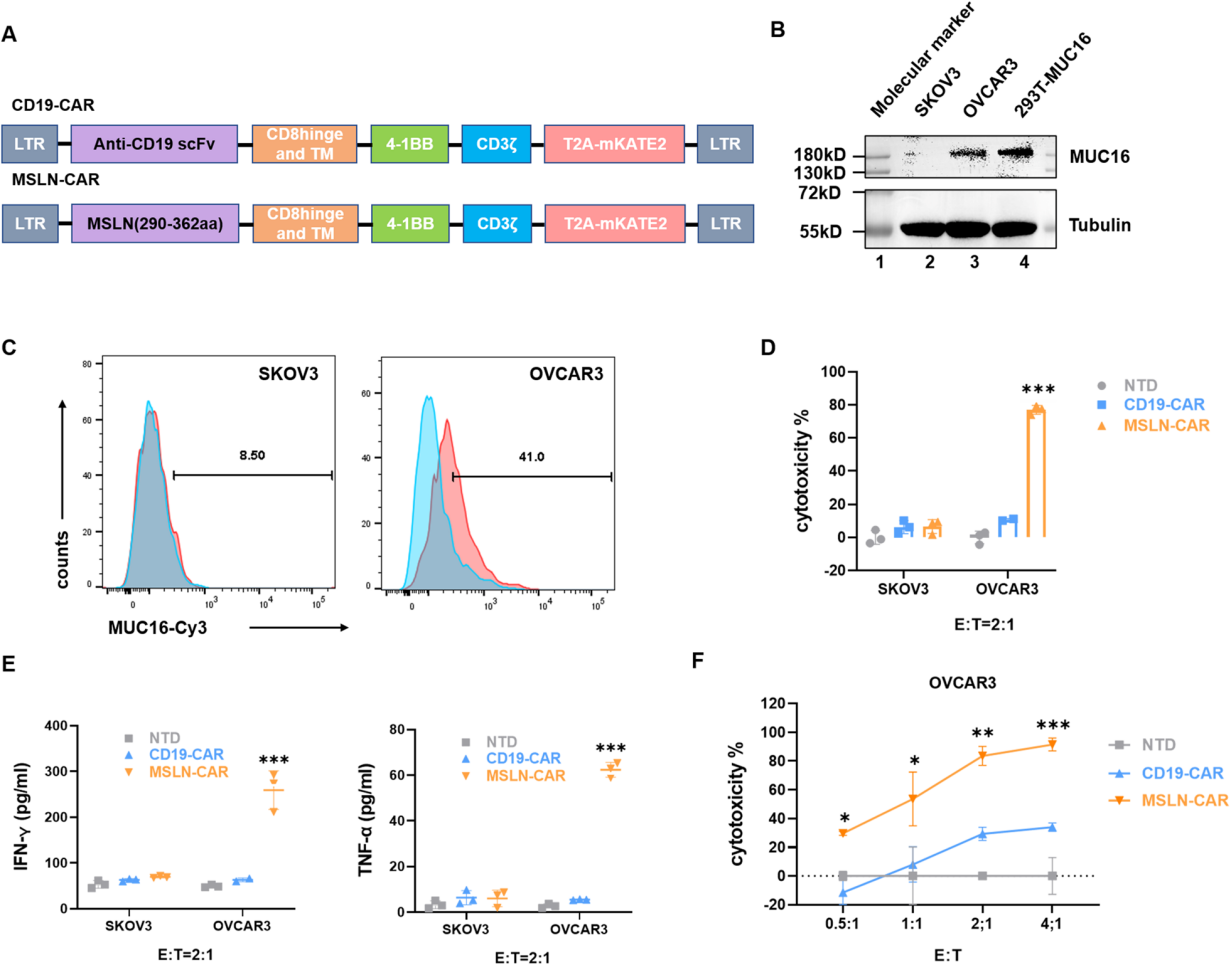
MSLN-CAR T cells can efficiently lyse MUC16-positive human ovarian cancer cells. **A** Schematic illustration of a CD19-CAR and a MSLN-CAR T vector, respectively. **B**, **C** Quantitative analysis of MUC16 protein expression levels in ovarian cells (SKOV3, OVCAR3) by Western blotting (**B**) and flow cytometry (**C**). Tubulin was used as a loading control. Lane 1 = molecular marker; Lanes 2 = SKOV3 cell lysate; Lanes 3 = OVCAR3 cell lysate; Lanes 4 = MUC16 overexpression in 293T cell lysate. B and C consist of three technical replicates. The pictures represent one example of three technical replicates. **D** The cytotoxicity of MSLN-CAR T cells on ovarian cancer cell lines was quantified using a luciferase assay. Primary human T cells transduced with the indicated lentiviruses were co-incubated with target cells expressing luciferase at an effector to target (E:T) ratio of 2:1 for 20 h. Three independent experiments were performed. **E** Quantification of IFN-γ (*left*) and TNF-α (*right*) release in response to coculture with CD19-CAR or MSLN-CAR T cells at an E:T ratio of 2:1, as measured by ELISA. Data are presented as the mean ± SD*, n* = 3. **F** CAR T cells were co-incubated with target cells expressing luciferase at varying effector to target (E:T) ratios for 20 h. **D**–**F** consist of three biologic replicates and three technical replicates. Statistics: two-tailed one-way ANOVA. The results are presented as the mean volume ± SD; *P < 0.05, **P < 0.01, ***P < 0.001 vs CD19-CAR or NTD

In order to develop a MUC16-targeted chimeric antigen receptor (CAR) based on ligand/receptor mesothelin, we constructed MSLN-CAR and evaluated its cytotoxic potential in a series of experiments, while control experiments were carried out in parallel using T cells that expressed an unrelated CD19-CAR (Fig. 1A). To begin with, we characterized the expression of MUC16 in ovarian cancer cell lines SKOV3 and OVCAR3 at the RNA and protein levels (Fig. 1B, Additional file 1: Figure S1A, B). As shown, MUC16/CA125 expression in OVCAR3 cells was markedly higher than in SKOV3 cells. Additionally, we performed flow cytometry analysis and immunofluorescence staining of live cells to find that OVCAR3 exhibited high surface expression of MUC16, whereas SKOV3 exhibited low surface expression (Fig. 1C, Additional file 1: Figure S1C). Next, we assessed the cytotoxic actions of MSLN-CAR T cells on OVCAR3 and SKOV3 cell lines using co-culture experiments. As shown, T cells transduced with MSLN-CAR demonstrated significant cytotoxicity compared to T cells expressing CD19-CAR (Fig. 1D). The cytotoxic actions of MSLN-CAR T cells on OVCAR3 and not SKOV3 cells is consistent with the cytokine secretion profiles in the respective treatment conditions, in contrast to the lack of effect observed in nontransduced cells (NTD) and negative control (CD19-CAR) treatment groups (Fig. 1E). Furthermore, it was observed that the dose-dependent effect of MSLN-CAR T on the specific killing of OVCAR3 cells, which differs from the non-specific cytotoxic effect of CD19-CAR (Fig. 1F).

### The cytotoxic effects of MSLN-CAR T cells on ovarian tumor cells is dependent on high MUC16 expression levels

**Fig. 2:**
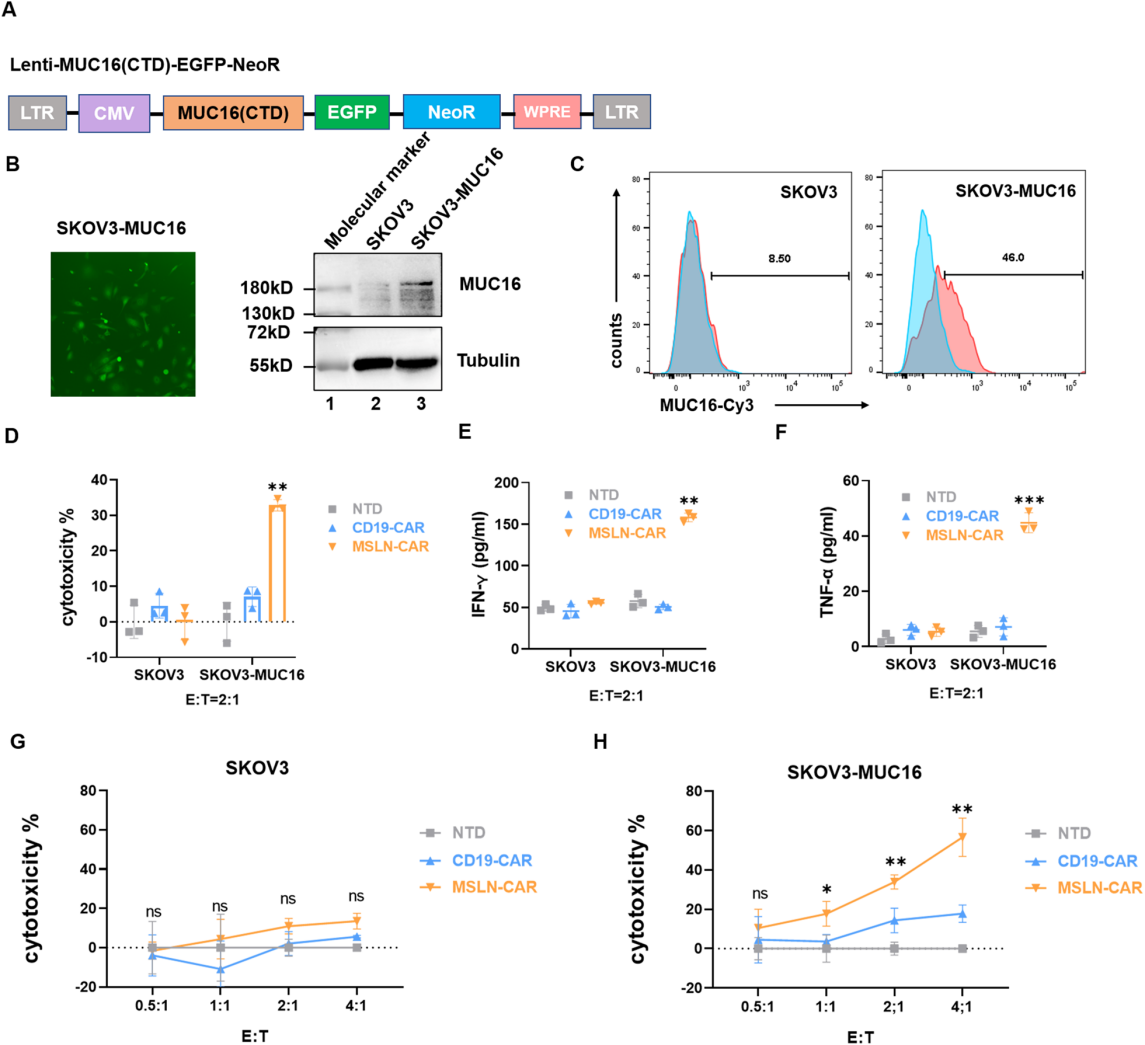
MSLN-CAR T cells target MUC16-overexpressing cells for cytotoxic killing. **A** Structure of the pLGNe-MUC16 expression plasmid used for overexpression assays. **B** The level of MUC16 protein overexpression in SKOV3 cell line (*left*) was evaluated by Western blot (*light*), with Tubulin was used as a loading control. Lane 1 = molecular marker; Lanes 2 = SKOV3 cell lysate; Lanes 3 = MUC16 overexpression in SKOV3 cell lysate. B and C consist of three technical replicates. The pictures represent one example of three technical replicates. **C** Flow cytometry was used to detect MUC16 expression on the surface of viable, SKOV3 cells. **D** Cytotoxicity of MSLN-CAR T cells following their co-culture with MUC16-overexpressing cells as tested with a luciferase-based assay. CAR T cells were incubated with luciferase-expressing target cells for 20 h at an effector to target (E:T) ratio of 2:1. **E**, **F** ELISA-based quantification of IFN-γ (**E**) and TNF-α (**F**) released in response to coculture with Mock or MSLN-CAR T cells at an E:T ratio of 2:1. **G**, **H** CAR T cells were co-incubated with target cells expressing luciferase at varying effector to target (E:T) ratios for 20 h. **D**–**H** consist of three biologic replicates and three technical replicates. Statistics: two-tailed one-way ANOVA. The results are presented as the mean volume ± SD; ns = not significant; *P < 0.05, **P < 0.01 vs CD19-CAR or NTD

To confirm that the cytotoxic impact of MSLN-CAR T cells on ovarian cancer cells was dependent on MUC16 expression, we asked if forced expression of MUC16 in SKOV3 cells could lead to their cytotoxic killing (Fig. 2A). To address this issue, we began by successfully overexpressing MUC16 in these cells, as confirmed by Western blotting, as well as by flow cytometry and immunofluorescence staining of live cells to confirm surface expression of MUC16 (Fig. 2B and C, Additional file 2: Figure S2). Next, we incubated MSLN-CAR T cells with MUC16-overexpressing SKOV3 cells and found that they were efficiently eliminated, in contrast to the negative control (control SKOV3 cells transfected with empty vector) (Fig. 2D). The study conducted by Reinartz et al. demonstrated that selective splicing or post-translational modifications may result in the expression of MUC16 on SKOV3 cells, which can be detected through western blot analysis, albeit expression levels are very low especially on the cell surface [35]. Compared with OVCAR3, the killing effect also reached only about 40%. However, the release of cytokines INF-γ and TNF-α also confirmed the specific cytotoxic effect of MSLN-CAR T against overexpressed cells (Fig. 2E, F). As a control experiment, neither MSLN-CAR T cells, CD19-CAR T cells or NTD treatment led to cytotoxic killing of SKOV3 cells (Fig. 2G). In contrast, the cytotoxicity of MSLN-CAR T cells against MUC16-overexpression SKOV3 cells increased in a concentration-dependent manner (Fig. 2H). While CD19-CAR T cells exhibited weak cytotoxicity against cells at high-efficiency target ratios, this difference was not statistically significant. Thus, our MSLN-CAR T cell approach shows specificity for MUC16 overexpression in ovarian cancer cells that drives their effective killing in vitro.

### MSLN-CAR T cells efficiently kill tumor stem-like cells

**Fig. 3:**
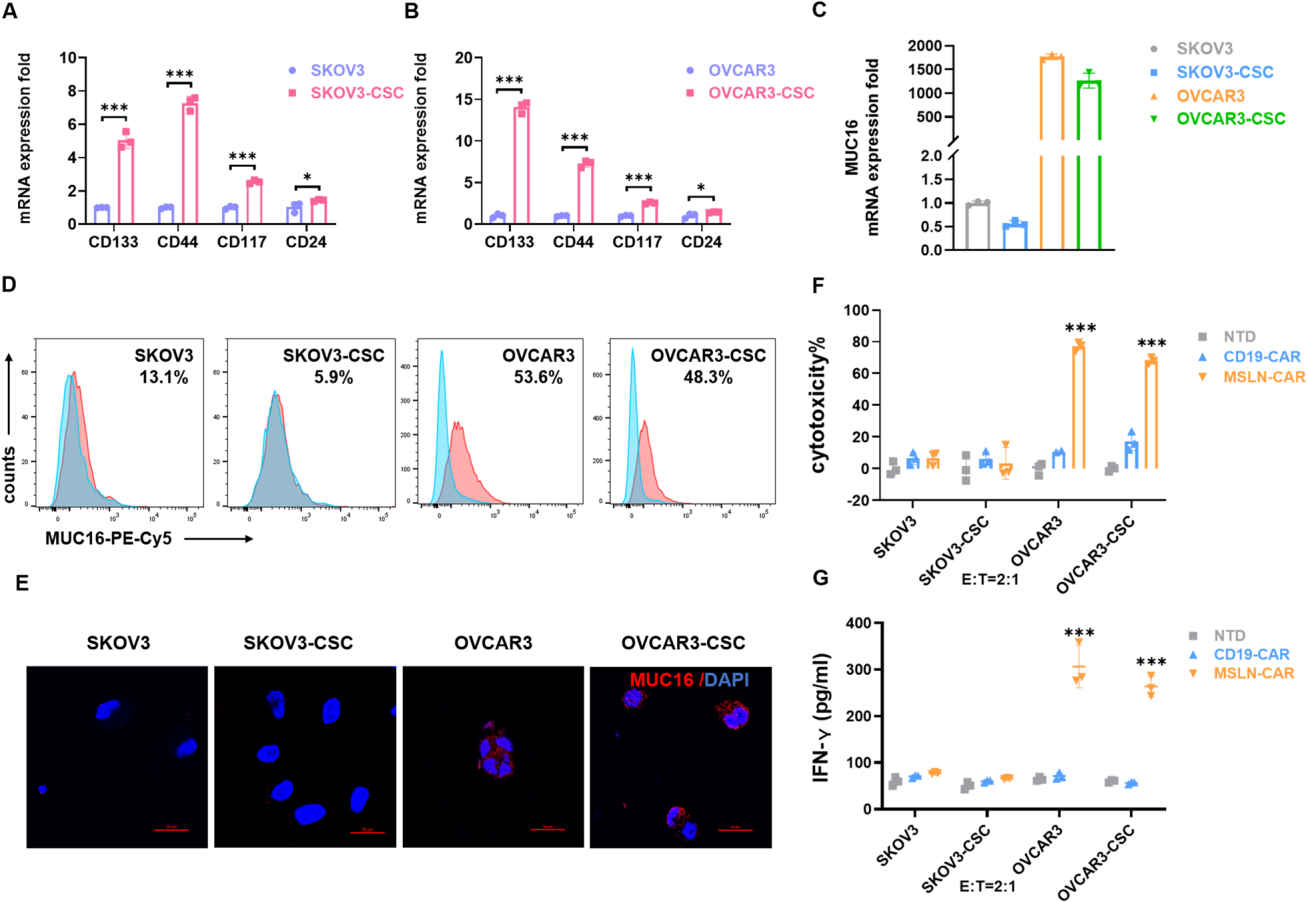
MSLN-CAR T cells efficiently kill cancer stem-like cells. **A**, **B** Quantification of RNA transcript levels for CD133, CD44, CD117 and CD24 in ovarian cells (SKOV3, OVCAR3) and their cancer stem cell derivations (SKOV3-CSC and OVCAR3-CSC). For statistical analysis of two groups an unpaired two sample t-test is used. Data were plotted and are shown as mean ± SD; * P < 0.05, *** P < 0.001. **C** Quantification of RNA transcripts for MUC16 in ovarian cells (SKOV3, OVCAR3) and their cancer stem cell derivations (SKOV3-CSC and OVCAR3-CSC) were determined by confocal imaging. **D**, **E** Both flow cytometry and immunofluorescence staining were used to stain live cells. **A**–**D** consist of three technical replicates. The pictures represent one example of three technical replicates. **F** The cytotoxicity of MSLN-CAR T cells on luciferase-expressing target cells was quantified after incubation for 20 h at an E:T ratio of 2:1. Three independent experiments were performed per condition. **G** ELISAs were used to detect IFN-γ release by T cells in coculture supernatants. **E** and **F** consist of three biologic replicates and three technical replicates. Statistics: two-tailed one-way ANOVA. Data are presented as the mean ± SD*, n* = 3; ***P < 0.001 vs CD19-CAR or NTD

Cancer stem-like cells (CSCs) play a significant role in the initiation, recurrent growth and metastatic invasion features of tumors, and also influence the effectiveness of tumor immunotherapies [36, 37]. To assess the cytotoxicity of MSLN-CAR T cells against ovarian cancer stem cells (OCSCs), we derived sphere cultures from SKOV3 and OVCAR3 cells by culturing these in stem cell medium supplemented with EGF, FGF, and B27 (Additional file 3: Figure S3A, see “Materials and methods” for details). Confirmation of “stemness” was achieved through characterization of the expression of stem cell markers CD133, CXCR4, and OCT4 by these CSCs (Fig. 3A, B). At the level of RNA expression, the expression of MUC16 in CSCs was consistent with that of the parental cell line (Fig. 3C). Live-cell staining was performed on OVCAR3-CSCs and parental cells, and flow cytometry and immunofluorescence confirmed their high expression levels of MUC16 on the cell membrane surface (Fig. 3D, E). The high expression of MUC16 was further supported by Western blot analysis (Additional file 3: Figure S3B). Upon confirmation in the OVCAR3-CSCs, co-culture with MSLN-CAR T cells resulted in effective cytotoxic killing (Fig. 3F), as evidenced by the significant secretion of IFN-γ and TNF-α into the culture media (Fig. 3G, Additional file 3: Figure S3C). In contrast, SKOV3 cells and SKOV3-CSCs expressed low MUC16 levels, a co-culture with MSLN-CAR T cells did not lead to their cytotoxic killing, nor significant cytokine release (Fig. 3F, G, Additional file 3: Figure S3C).

### MSLN-CAR T cells showed effective and persistent antitumor activity against OVCAR3 xenografts in mice

**Fig. 4:**
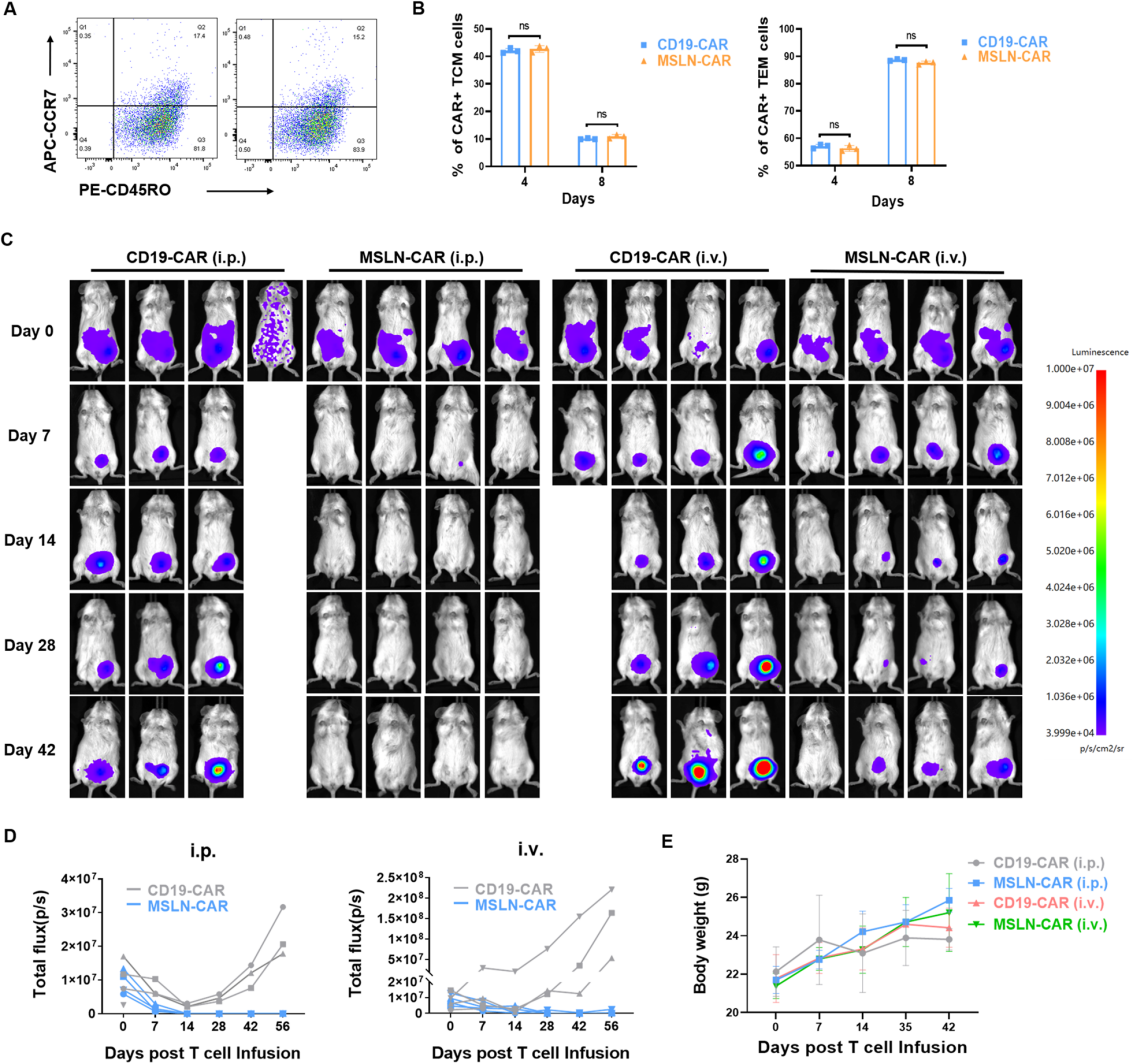
Antitumor effects of MSLN-CAR T cells on ovarian cancer cell xenografts in vivo. **A** Gating strategy of flow cytometry to discriminate TN, TCM, TEM, and TEMRA cells. **B** Flow cytometry was used to analyze the percentages of Tcm and TemT cell subsets in various CARs, with results reported as mean ± SD, ns means no significance. **C** NCG mice were intraperitoneally injected with 5 × 10^5^ OVCAR3 cells and imaged on the fifth day. Next, BLI of tumor progression of representative mice receiving intraperitoneally injected or intravenously (tail vein) injected MSLN-CAR T cells, compared to control (CD19-CAR T cell treated) mice. **D** Quantification of tumor bioluminescence levels at different time points (*n* = 4 mice per group).** (E)** Quantification of body weights of mice receiving receiving intraperitoneally injected or intravenously (tail vein) injected MSLN-CAR T cells or CD19-CAR T cells. The experiments were repeated twice, independently, with similar results

The optimal CD4/CD8 ratio of chimeric antigen receptor (CAR) T cells is approximately 1:1, which confers a favorable advantage for eliciting an effective anti-tumor response within the host organism [38, 39]. When analyzed on Day 4 or on Day 8 following CD3/28 stimulation, MSLN-CAR T cells were not significantly different in their CD4 and CD8a expression profiles when compared with nontransduced T (NTD) cells, or with control (CD19-CAR T) cells (Additional file 4: Figure S4A). Furthermore, the central/effector memory phenotype of MSLN-CAR T cells was found to be similar to that of CD19-CAR T cells, as assessed by the expression of CD45RO and CCR7 (Fig. 4A, B). Following antigen stimulation, we investigated the expression of T cell depleting factors and found no significant difference in PD-1 and TIM-3 expression on CAR T cells after co-culture with target cells. Interestingly, there was a slight but statistically significant decrease in LAG3 expression (Additional file 4: Figure S4B).

In order to evaluate the in vivo anti-tumor activity of MSLN-CAR T cells, we established a xenograft ovarian cancer tumor model through intraperitoneal injection of OVCAR3 cells into NCG mice. In this experiment, such mice develop significant ascites and multiple nodular peritoneal tumors 5 weeks after OVCAR3 cell injections [40]. We were interested to explore whether MSLN CAR-T injections in this model, particularly to determine if tail vein injections or intraperitoneal injections of CAR-T cells could effectively shrink tumors, or not; because tumor targeting by CAR-T cell therapies is currently limited [41]. As a negative control, we performed parallel experiments in which mice with tumors were injected with CD19-CAR T cells. As shown, compared to the CD19-CAR group, both intraperitoneal delivery and tail vein injections of MSLN-CAR T cells markedly suppressed tumor growth. Notably, mice that received intraperitoneal injections exhibited complete regression of tumors by day 42, in contrast to mice that received tail vein injections of MSLN-CAR T cells (Fig. 4C, D). In contrast, mice in the control group injected with CD19-CAR T cells featured heavier tumor burdens and exhibited a trend toward weight loss, whereas the experimental group did not show such effects (Fig. 4E).

**Fig. 5:**
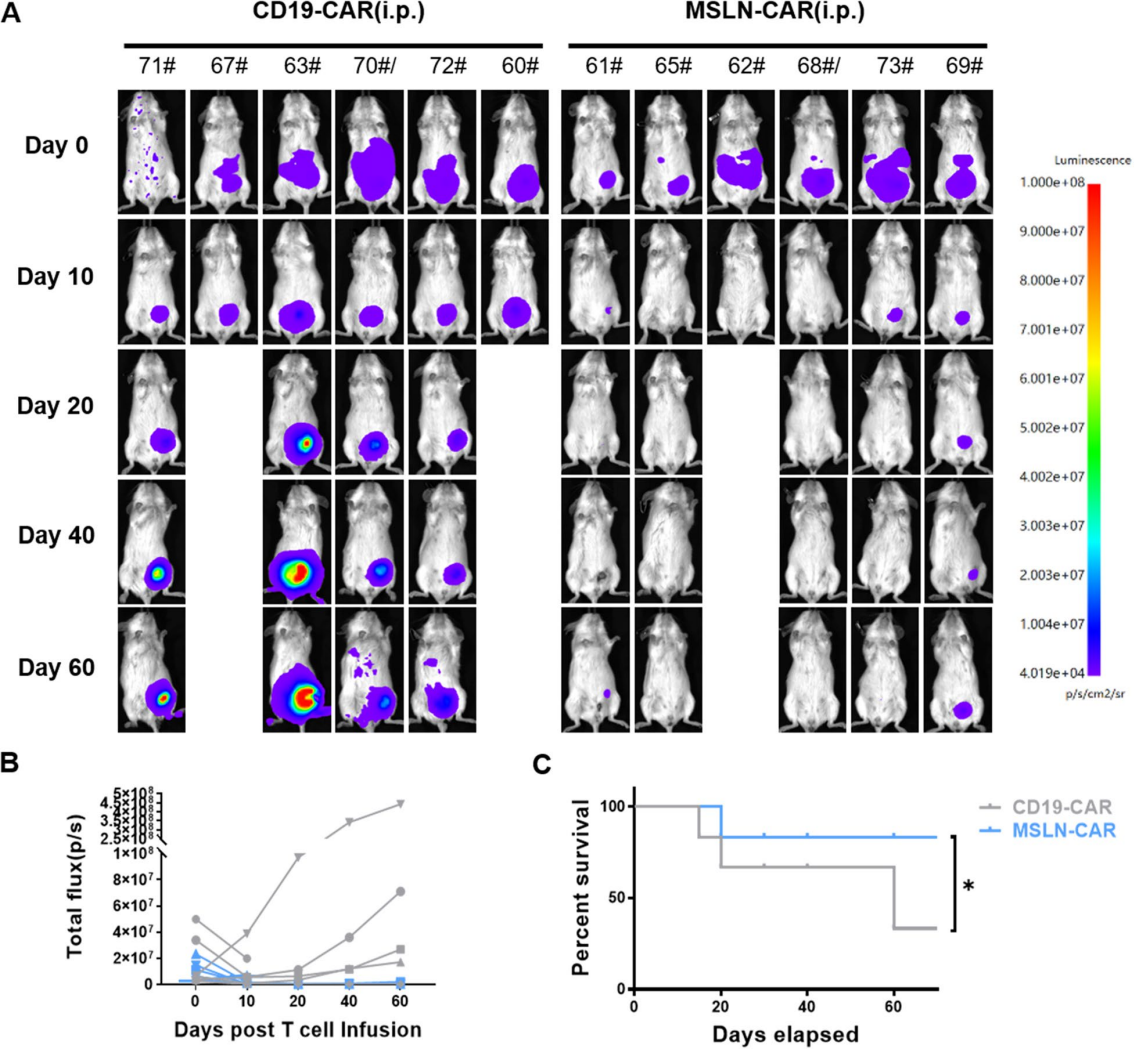
Prolonged survival of NCG mice with ovarian cancer cell xenograft tumors following intraperitoneal infusion of MSLN-CAR T cells. **A** Human ovarian tumor OVCAR3 cells were injected intraperitoneally into mice, following which 5 × 10^6^ of either CD19-CAR T cells or MSLN-CAR T cells were injected intraperitoneally. Following treatment with the respective CAR T cells, tumor burden was monitored at the indicated time points using bioluminescent imaging. **B** Quantification of tumor bioluminescence levels at different time points (*n* = 6 mice per group). **C** Kaplan–Meier survival curve of NCG mice treated i.p. with CD19-CAR or MSLN-CAR T cells. The experiments were repeated twice, independently, with similar results. Statistical analysis by two-tailed log rank test; n.d., not detected; *P < 0.05

Next, we investigated whether the anti-tumor effects of intraperitoneal injection of MSLN-CAR T cells into NCG mice with ovarian cancer xenografts model were of prolonged impact. In this experiment, we first injected NCG mice with OVCAR3 tumor cells and kept the mice for five days, following which MSLN-CAR T cells were intraperitoneally infused and the effects on tumors were analyzed. As shown, based on BLI imaging and overall survival assessment (Fig. 5A, B), we find that intraperitoneal administration of MSLN-CAR T cells suppressed tumor growth and resulted in prolonged survival of these mice compared to control mice treated with CD19-CAR T cells (Fig. 5C).

### The safety of MSLN-CAR was assessed in vitro and in vivo

**Fig. 6:**
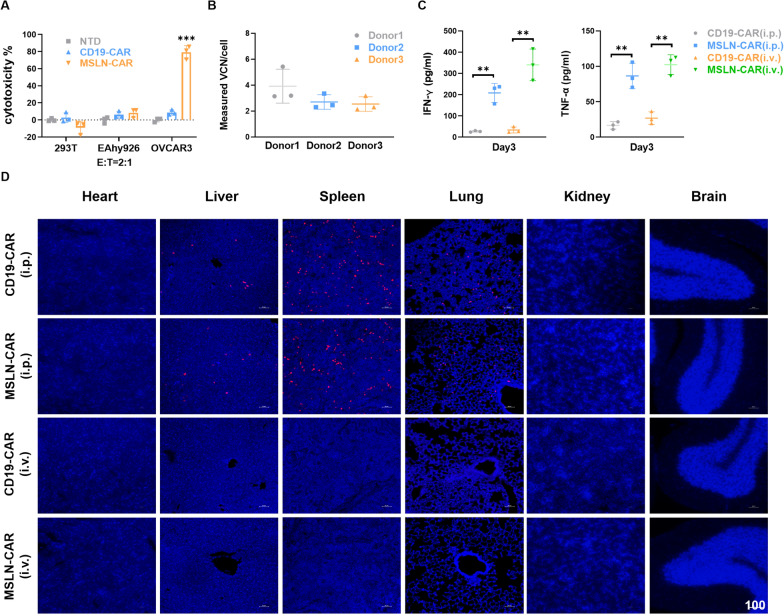
Specificity and safety of MSLN-CAR T cells. **A** The cytotoxicity of MSLN-CAR T cells on luciferase-expressing target cells was quantified after incubation for 20 h at an E:T ratio of 2:1. Three independent experiments were performed per condition. Statistics: two-tailed one-way ANOVA. Data are presented as the mean ± SD*, n* = 3; ***P < 0.001 vs CD19-CAR or NTD. **B** Quantification of retroviral vector copy number (VCN) in transduced T cells (CAR). Measured VCN per cell of MSLN-CAR T cells derived from three healthy donors above. Cells were assessed 96 h post transduction and the transduction MOI and efficiency were the same as the above. The experiment was conducted using three biologic replicates, each one including three technical replicates. **C** Levels of IFN-γ (left), and TNF-α (right) were measured by ELISA on serum collected from peripheral blood on three days after immune effector cell infusion. For statistical analysis of two groups an unpaired two sample t-test is used. Data are presented as the mean ± SD*, n* = 3. **P < 0.01 vs CD19-CAR. **D** Designated organs were analyzed after fluorescent staining of immune tissues on day 5 after intraperitoneal or intravenous administration of CAR T cells. Representative confocal microscopy images of CD3^+^ T cells in major organs (heart, liver, spleen, lung, kidney, brain) (n = 3 mice/group). Scale bar = 100 μm. The experiments were repeated twice, independently, with similar results

MUC16 is expressed not only in tumor cells, but also in normal tissues, particularly in glands and epithelial cells [42, 43]. Therefore, we assessed the safety of MSLN-CAR T cell treatment by evaluating their cytotoxicity towards non-cancerous cell lines. We found low levels of MUC16 protein expression in HEK-293 T and EAhy926 cells that were insufficient to trigger the activation of MSLN-CAR T cells (Fig. 6A). CAR T cells derived from healthy donors were collected and directly analyzed for vector copy number (VCN) validation experiments. The VCN of the MSLN-CAR T cell products were approximately 5, 3, and 3 copies for Donor 1, 2 and 3, respectively (Fig. 6B).

In order to further assess the safety of MSLN-CAR T cells, we conducted experiments using additional mice to investigate the impact of MSLN-CAR T cells on the normal organs of the mice. In the course of immune effector cell treatment, mouse serum was collected on day 3 post CAR-T infusion to assess the levels of cytokines IFN-γ and TNF-α release (Fig. 6C). The treatment group exhibited a significant increase in cytokine levels compared to the control CAR group (CD19-CAR). Subsequently, we observed that after intraperitoneal and tail vein injections of MSLN-CAR T cells, CD3 immunohistochemical staining revealed organ distribution in the intraperitoneal injection group, while in the intravenous treatment group, a small amount number of organs with a rich blood supply, such as liver and lung were observed to contain numerous cells which were CD3 immunostained (Fig. 6D). Following HE staining, it was confirmed that there was no significant tissue damage or structural changes in the main organs (heart, liver, spleen, lung, kidney and brain) of mice (Additional file 5: Figure S5). This result indicates that injections of MSLN-CAR T cells do not cause organ damage in mice, at least in the timepoints that we have investigated here.

## Discussion

The aberrant expression of MUC16 (CA125) is often implicated in various diseases, and there has been growing interest in the development of targeting drugs for treating cancer cells which overexpress MUC16 [9, 19, 44]. One significant challenge for current antibody-based cancer therapies that target MUC16-expressing tumors is the fact that the majority of the extracellular domain of MUC16 is cleaved and secreted, limiting the efficacy of MUC16 as a target antigen for ovarian cancer [5, 40]. To date, the majority of reported monoclonal antibodies targeting MUC16 have been shown to bind to epitopes present on the glycoprotein shed CA-125 component. The development of chimeric antigen receptors (CARs) specifically binding to extracellular domains of the antigen-retained fraction (MUC-CD) may significantly overcome the limitations of MUC16 as a target for adoptive cellular immunotherapy. Here, we have constructed CAR T cells based on mesothelin polypeptide sequences that bind MUC16 to effectively eliminate MUC16-expressing ovarian cancer tumor cells and stem cells in vitro, as well as to eliminative ovarian tumor xenografts in vivo. Our study offers a highly specific approach through which to target MUC16 using ligand-receptor engineered CAR T cell therapy, together with its apparently low organ toxicity as evaluated in our mouse xenograft models.

The interaction between MUC16 (CA125) and mesothelin is believed to facilitate tumor implantation and metastasis [5, 45, 46]. Specifically, the binding of MUC16 (CA125) to mesothelin downregulates DKK1 (Dickkopf-1, an inhibitor of the WNT signaling pathway) through the SGK3/FOXO3 signaling pathway, thereby promoting migration [11]. Hence, this mechanism is considered a potential contributor to peritoneal metastasis in ovarian tumors. Interestingly, the membrane-bound form of MUC16 may have a higher affinity for mesothelin than the secreted form, as demonstrated, even in the presence of peritoneal fluid [13]. This characteristic offers the potential for the development of chimeric antigen receptors (CARs) that specifically bind to the extracellular domain of the MUC16 antigen retention portion (MUC-CD). Zhao et al. colleagues conducted a partial in vitro study on the construction of CAR T cells using the N-terminal fragment of mesothelin, and results are largely consistent with our current work [26]. However, Zhao and coworkers reported that systemic infusion of CAR T cells that were constructed based on the N-terminus of mesothelin did not eliminate tumor cells in mice while, in contrast, our MSLN-CAR T cells were effective at shrinking ovarian cancer cell tumors in NCG mice. We interpreted these results to indicate that both the expression level and intensity of MUC16 on the surface of tumor cells was crucial considerations when design an effective MUC16 CAR T cell therapy, such that ovarian cancer cells that expressed low levels of cell-surface MUC16 might not be efficiently targeted by targeting CAR T cells for elimination. Consistent with this, we performed live cell staining to find that the surface expression of MUC16 in SKOV3 cells was significantly lower, compared to OVCAR3 cells. These findings are consistent with previous studies and offer an explanation to the apparent discrepancy between the work of Zhao and colleagues and our current study [47, 48]. Further confirmation of our hypothesis was provided by our experiments that showed that MSLN-CAR T cells could lyse SKOV3 cells that overexpressed MUC16. Thus, the specificity of MSLN-CAR T cells for ovarian cancer cells is related to such cells expressing high levels of MUC16.

Previous work has demonstrated the effectiveness of MSLN-CAR T to effectively kill MUC16-expressing cells in vitro, however challenges remain as to its effectiveness in vivo, such as its low homing rate. Current approaches involve local injection to administer CAR T cells and to enhance their infiltration and proximity to solid tumors in patients, including to tumors in the brain, breast, pleura, and liver [49–51]. Given that the majority of ovarian cancer metastases occur in the peritoneal cavity, and that peritoneal perfusion chemotherapy has a well-established history of success as a therapeutic approach; it is perhaps unsurprising that the efficacy of intraperitoneal injections of CAR T in mouse models also shows that this is an effective treatment approach [40, 52]. Guided by this insight, we assessed the effects of systemic and local administration of MSLN-CAR T cells on tumor growth in mice and found that both intraperitoneal and tail vein injections of MSLN-CAR T cells could suppress the growth and peritoneal dissemination of OVCAR3 xenografts. Furthermore, the tumors in mice treated with intraperitoneal injection continued to regress after 42 days, and a significant extension in the survival period of tumor-bearing mice was observed. Furthermore, we also found that localized infusion of MSLN-CAR T cells could mitigate the limitations imposed by the homing difficulties of CAR T cells. Our results are consistent with findings by others that indicate that intraperitoneal injections are effective as a primary treatment approach for ovarian cancer CAR-T.

Due to their unlimited self-renewal and tumorigenic potential, ovarian cancer stem cells (OCSCs) play a crucial role in tumor formation and metastasis [53, 54]. One notable attribute of OCSCs is their capacity to persist and multiply even in the absence of adhesion to peritoneal sites or onto abdominal organs, or both. In addition, their poor circulation as tumor cells also facilitates it resistance against chemotherapy [55, 56]. The expression of MUC16 in OCSC has been identified as the primary source of tumor metastasis and recurrence [57, 58], and our study has confirmed the ability of MSLN-CAR T cells to recognize and eliminate CSCs derived from ovarian cancer cell lines that express MUC16. Our results highlight the potential for MSLN-CAR T cells as an effective therapeutic strategy for eliminating CSCs.

The immunogenicity and targeted toxicity of CAR structures remains an important consideration in the development of treatments using this approach [59]. The immunogenicity of CAR T cells can trigger an immune response against CAR, leading to rejection, deletion, and clearance of CAR T cells, which is also a crucial factor affecting the long-term persistence of CAR T cells in the body [60]. Previous studies have indicated that the upregulation of CD28 or 4-1BB signals in CAR structures, as well as high expression of CAR molecules in T cells, result in increased cytotoxicity. However, this also leads to the expression of exhaustion markers in T cells, such as PD-1 and TIM-3 [61, 62]. Further, there is an increased risk of oncogenesis if the vector copy number (VCN) per cell is high. It is important to assess VCN as FDA recommends release of CAR-T finished product, particularly when considering clinical use. Our results showed that the vector copy number of MSLN-CAR T cells products was similar to that approved by the FDA. To determine if this issue was prevalent in our CAR T approach, we co-cultured MSLN-CAR T cells with OVCAR3 cells and found that it did not lead to significant depletion of CAR T cells. Consistent with the findings of Zhao et al., our results also suggest that CARs constructed based on mesothelin may delay T cell activation and prevent excessive activation-induced T cell exhaustion [26, 63]. In our in vivo experiments, we found that neither systemic nor local infusion methods employed resulted in significant pathological changes in the vital organs of the mice. We interpret these results to indicate that CAR T cells prepared with a ligand-binding domain of MUC16 based on mesothelin may potentially evade host immune responses to scFv and maintain anti-tumor activity. However, due to species differences between humans and mice, as well as the stable expression of CAR-T cells that may result in potentially life-threatening on-target tumor effects, it is necessary to conduct future studies in non-human primates and other animal models. These studies should focus on safety and immunogenicity in preclinical settings.

## Conclusions

This study demonstrates that mesothelin-based CAR T cells effectively target ovarian cancer cells and their stem cells for killing in vitro, and is potent as an anti-tumor agent when administered systemically or locally in a xenograft mouse model of ovarian cancer. Our findings showcase the potential for CAR T-cell therapies based on mesothelin that can treat ovarian cancer and other MUC16-positive malignancies. This technology has been granted a national patent (Patent No. ZL 202210507054.1).

### Supplementary Information

**Additional file 1: Figure S1.** The expression of MUC16 in ovarian cells (SKOV3, OVCAR3).

**Additional file 2: Figure S2.** Live cell immunofluorescence detectionof MUC16 overexpression in SKOV3 ovarian cells.

**Additional file 3: Figure S3.** Identification of ovariancancer stem-like cells.

**Additional file 4: Figure S4.** Detection of CD19-CAR and MSLN-CAR T cell typing and expression levels of depletion factors.

**Additional file 5: Figure S5.** Infusion of MSLN-CAR T cells does not cause evident toxicity.

**Additional file 6: Table S1.** STR Profile of Cell Lines.

**Additional file 7: Table S2.** The primer sequences.

**Additional file 8: Table S3.** Antibodies used in the study.

## Data Availability

The data generated in this study are available within the article and its Supplementary Data Files or upon request from the corresponding author.

## Abbreviations

CAR-T cells: Chimeric antigen receptor-T cells
MSLN: Mesothelin
MUC16: Mucin-16 (CA125, Cancer antigen 125)
OC: Ovarian cancer
IF: Immunofluorescence
CSCs: Cancer stem cells
EGFR: Epidermal growth factor receptor
BiTE: Bispecific T-cell engagers
ADC: Antibody–drug conjugates
scFv: Single-chain antibody fragments
NTD: Nontransduced
IVIS: In vivo imaging software
PBMC: Peripheral blood mononuclear cells
IFN-γ: Interferon γ
TNF-α: Tumor necrosis factor-α
DAPI: Diamidino-2-phenylindole
PD-1: Programmed cell death 1
TIM-3: T cell immunoglobulin domain and mucin domain-3
LAG3: Lymphocyte activation gene 3
VCN: Vector copy number

## References

[1] Hagimori M, Kato N, Orimoto A (2023). Development of triple-negative breast cancer-targeted liposomes with MUC16 binding peptide ligand in triple-negative breast cancer cells. J Pharm Sci.

[2] Matulonis UA, Sood AK, Fallowfield L (2016). Ovarian cancer. Nat Rev Dis Primers.

[3] Marimuthu S, Lakshmanan I, Muniyan S (2022). MUC16 promotes liver metastasis of pancreatic ductal adenocarcinoma by upregulating NRP2-associated cell adhesion. Mol Cancer Res.

[4] Chaudhary S, Appadurai MI, Maurya SK (2023). MUC16 promotes triple-negative breast cancer lung metastasis by modulating RNA-binding protein ELAVL1/HUR. Breast Cancer Res.

[5] Felder M, Kapur A, Gonzalez-Bosquet J (2014). MUC16 (CA125): tumor biomarker to cancer therapy, a work in progress. Mol Cancer.

[6] Nunez J, de la Espriella R, Minana G (2021). Antigen carbohydrate 125 as a biomarker in heart failure: a narrative review. Eur J Heart Fail.

[7] Argueso P, Spurr-Michaud S, Russo CL (2003). MUC16 mucin is expressed by the human ocular surface epithelia and carries the H185 carbohydrate epitope. Invest Ophthalmol Vis Sci.

[8] Matte I, Garde-Granger P, Bessette P (2019). Ascites from ovarian cancer patients stimulates MUC16 mucin expression and secretion in human peritoneal mesothelial cells through an Akt-dependent pathway. BMC Cancer.

[9] Crawford A, Haber L, Kelly MP (2019). A Mucin 16 bispecific T cell-engaging antibody for the treatment of ovarian cancer. Sci Transl Med.

[10] Wang Q, Ma X, Wu H (2022). Oncolytic adenovirus with MUC16-BiTE shows enhanced antitumor immune response by reversing the tumor microenvironment in PDX model of ovarian cancer. Oncoimmunology.

[11] Huo Q, Xu C, Shao Y (2021). Free CA125 promotes ovarian cancer cell migration and tumor metastasis by binding Mesothelin to reduce DKK1 expression and activate the SGK3/FOXO3 pathway. Int J Biol Sci.

[12] Reynolds IS, Fichtner M, McNamara DA (2019). Mucin glycoproteins block apoptosis; promote invasion, proliferation, and migration; and cause chemoresistance through diverse pathways in epithelial cancers. Cancer Metastasis Rev.

[13] Gubbels JA, Belisle J, Onda M (2006). Mesothelin-MUC16 binding is a high affinity, N-glycan dependent interaction that facilitates peritoneal metastasis of ovarian tumors. Mol Cancer.

[14] Faust JR, Hamill D, Kolb EA (2022). Mesothelin: an immunotherapeutic target beyond solid tumors. Cancers (Basel).

[15] Kaneko O, Gong L, Zhang J (2009). A binding domain on mesothelin for CA125/MUC16. J Biol Chem.

[16] Gregory SN, Sarvestani AL, Ryan CE (2023). Oregovomab plus chemo in newly diagnosed patients with advanced epithelial ovarian cancer following optimal debulking surgery (FLORA-5/GOG-3035). Ann Surg Oncol.

[17] Koneru M, O'Cearbhaill R, Pendharkar S (2015). A phase I clinical trial of adoptive T cell therapy using IL-12 secreting MUC-16(ecto) directed chimeric antigen receptors for recurrent ovarian cancer. J Transl Med.

[18] Radhakrishnan P, Mohr AM, Grandgenett PM (2013). MicroRNA-200c modulates the expression of MUC4 and MUC16 by directly targeting their coding sequences in human pancreatic cancer. PLoS ONE.

[19] Shah A, Chaudhary S, Lakshmanan I (2023). Chimeric antibody targeting unique epitope on onco-mucin16 reduces tumor burden in pancreatic and lung malignancies. NPJ Precis Oncol.

[20] Chen W, Yuan Y, Jiang X (2020). Antibody and antibody fragments for cancer immunotherapy. J Control Release.

[21] Safarzadeh Kozani P, Naseri A, Mirarefin SMJ (2022). Nanobody-based CAR-T cells for cancer immunotherapy. Biomark Res.

[22] Camviel N, Wolf B, Croce G (2022). Both APRIL and antibody-fragment-based CAR T cells for myeloma induce BCMA downmodulation by trogocytosis and internalization. J Immunother Cancer.

[23] Majzner RG, Mackall CL (2018). Tumor antigen escape from CAR T-cell therapy. Cancer Discov.

[24] Ramirez-Chacon A, Betriu-Mendez S, Bartolo-Ibars A (2022). Ligand-based CAR-T cell: Different strategies to drive T cells in future new treatments. Front Immunol.

[25] Branella GM, Spencer HT (2021). Natural receptor- and ligand-based chimeric antigen receptors: strategies using natural ligands and receptors for targeted cell killing. Cells.

[26] Zhao H, Wu L, Dai J (2023). Ligand-based adoptive T cell targeting CA125 in ovarian cancer. J Transl Med.

[27] Guo J, He S, Zhu Y (2021). Humanized CD30-targeted chimeric antigen receptor T cells exhibit potent preclinical activity against Hodgkin’s lymphoma cells. Front Cell Dev Biol.

[28] Wei W, Ma H, Yang D (2023). SECTM1-based CAR T cells enriched with CD7-low/negative subsets exhibit efficacy in CD7-positive malignancies. Blood Adv.

[29] Yang D, Cheng D, Tu Q (2018). HUWE1 controls the development of non-small cell lung cancer through down-regulation of p53. Theranostics.

[30] Christodoulou I, Rahnama R, Ravich JW (2021). Glycoprotein targeted CAR-NK cells for the treatment of SARS-CoV-2 infection. Front Immunol.

[31] Wang S, Wei W, Yuan Y (2023). Chimeric antigen receptor T cells targeting cell surface GRP78 efficiently kill glioblastoma and cancer stem cells. J Transl Med.

[32] Li Y, Hermanson DL, Moriarity BS (2018). Human iPSC-derived natural killer cells engineered with chimeric antigen receptors enhance anti-tumor activity. Cell Stem Cell.

[33] Chen X, Li X, Wang X (2019). MUC16 impacts tumor proliferation and migration through cytoplasmic translocation of P120-catenin in epithelial ovarian cancer cells: an original research. BMC Cancer.

[34] Fan J, Yu Y, Yan L (2023). GAS6-based CAR-T cells exhibit potent antitumor activity against pancreatic cancer. J Hematol Oncol.

[35] Sharma SK, Mack KN, Piersigilli A (2022). ImmunoPET of ovarian and pancreatic cancer with AR9.6, a novel MUC16-targeted therapeutic antibody. Clin Cancer Res.

[36] Huang T, Song X, Xu D (2020). Stem cell programs in cancer initiation, progression, and therapy resistance. Theranostics.

[37] Yuan S, Stewart KS, Yang Y (2022). Ras drives malignancy through stem cell crosstalk with the microenvironment. Nature.

[38] Gyobu H, Tsuji T, Suzuki Y (2004). Generation and targeting of human tumor-specific Tc1 and Th1 cells transduced with a lentivirus containing a chimeric immunoglobulin T-cell receptor. Cancer Res.

[39] Moeller M, Kershaw MH, Cameron R (2007). Sustained antigen-specific antitumor recall response mediated by gene-modified CD4+ T helper-1 and CD8+ T cells. Cancer Res.

[40] Chekmasova AA, Rao TD, Nikhamin Y (2010). Successful eradication of established peritoneal ovarian tumors in SCID-Beige mice following adoptive transfer of T cells genetically targeted to the MUC16 antigen. Clin Cancer Res.

[41] Lahiri A, Maji A, Potdar PD (2023). Lung cancer immunotherapy: progress, pitfalls, and promises. Mol Cancer.

[42] Olson MT, Wojtynek NE, Talmon GA (2020). Development of a MUC16-targeted near-infrared fluorescent antibody conjugate for intraoperative imaging of pancreatic cancer. Mol Cancer Ther.

[43] Mi Y, Huang Y, Deng J (2018). The enhanced delivery of salinomycin to CD133(+) ovarian cancer stem cells through CD133 antibody conjugation with poly(lactic-co-glycolic acid)-poly(ethylene glycol) nanoparticles. Oncol Lett.

[44] Liu JF, Moore KN, Birrer MJ (2016). Phase I study of safety and pharmacokinetics of the anti-MUC16 antibody-drug conjugate DMUC5754A in patients with platinum-resistant ovarian cancer or unresectable pancreatic cancer. Ann Oncol.

[45] Aithal A, Rauth S, Kshirsagar P (2018). MUC16 as a novel target for cancer therapy. Expert Opin Ther Targets.

[46] Schuster H, Peper JK, Bosmuller HC (2017). The immunopeptidomic landscape of ovarian carcinomas. Proc Natl Acad Sci U S A.

[47] Babeker H, Ketchemen JP, Annan Sudarsan A (2022). Engineering of a fully human anti-MUC-16 antibody and evaluation as a PET imaging agent. Pharmaceutics.

[48] Theriault C, Pinard M, Comamala M (2011). MUC16 (CA125) regulates epithelial ovarian cancer cell growth, tumorigenesis and metastasis. Gynecol Oncol.

[49] Cherkassky L, Hou Z, Amador-Molina A (2022). Regional CAR T cell therapy: an ignition key for systemic immunity in solid tumors. Cancer Cell.

[50] Li G, Guo J, Zheng Y (2021). CXCR5 guides migration and tumor eradication of anti-EGFR chimeric antigen receptor T cells. Mol Ther Oncolytics.

[51] Pan K, Farrukh H, Chittepu V (2022). CAR race to cancer immunotherapy: from CAR T, CAR NK to CAR macrophage therapy. J Exp Clin Cancer Res.

[52] Li T, Wang J (2020). Therapeutic effect of dual CAR-T targeting PDL1 and MUC16 antigens on ovarian cancer cells in mice. BMC Cancer.

[53] Parte SC, Batra SK (2018). and Kakar S S Characterization of stem cell and cancer stem cell populations in ovary and ovarian tumors. J Ovarian Res.

[54] Kenda Suster N, Virant-Klun I (2019). Presence and role of stem cells in ovarian cancer. World J Stem Cells.

[55] Dontu G, Abdallah WM, Foley JM (2003). In vitro propagation and transcriptional profiling of human mammary stem/progenitor cells. Genes Dev.

[56] Mo L, Bachelder RE, Kennedy M (2015). Syngeneic murine ovarian cancer model reveals that ascites enriches for ovarian cancer stem-like cells expressing membrane GRP78. Mol Cancer Ther.

[57] Zhang H, Yang Y, Wang Y (2015). Relationship of tumor marker CA125 and ovarian tumor stem cells: preliminary identification. J Ovarian Res.

[58] Abubaker K, Latifi A, Luwor R (2013). Short-term single treatment of chemotherapy results in the enrichment of ovarian cancer stem cell-like cells leading to an increased tumor burden. Mol Cancer.

[59] Lamers CH, Klaver Y, Gratama JW (2016). Treatment of metastatic renal cell carcinoma (mRCC) with CAIX CAR-engineered T-cells-a completed study overview. Biochem Soc Trans.

[60] Huang L, Li J, Yang J (2022). Safety and efficacy of humanized versus murinized CD19 and CD22 CAR T-cell cocktail therapy for refractory/relapsed B-cell lymphoma. Cells.

[61] Cappell KM, Kochenderfer JNA (2021). comparison of chimeric antigen receptors containing CD28 versus 4–1BB costimulatory domains. Nat Rev Clin Oncol.

[62] Rodriguez-Marquez P, Calleja-Cervantes ME, Serrano G (2022). CAR density influences antitumoral efficacy of BCMA CAR T cells and correlates with clinical outcome. Sci Adv.

[63] Qian C, Cao X (2021). Reversing the mitochondrial stress-induced exhaustion of CD8(+) T cells for improving cancer immunotherapy. Cell Mol Immunol.

