# Supplementary material for: Mesothelin-based CAR-T cells exhibit potent antitumor activity against ovarian cancer
Source: J Transl Med. 2024 Apr 18;22:367. doi: 10.1186/s12967-024-05174-y (PMC11025286; doi:10.1186/s12967-024-05174-y)
Supplement: Supplementary file 1 — Additional file 1: Figure S1. The expression of MUC16 in ovarian cells (SKOV3, OVCAR3). [file 12967_2024_5174_MOESM1_ESM.pdf]

Mesothelin-based CAR-T cells exhibit potent antitumor activity against ovarian cancer

Jing Guo[1], Xiaozhu Zeng[1], Yongjie Zhu[1], Dong Yang[1] and Xudong Zhao[1]*

[1]Department of Targeting Therapy & Immunology and Laboratory of Animal Tumor Models, Cancer Center and State Key Laboratory of Respiratory Health and Multimorbidity and Frontiers Science Center for Disease-related Molecular Network, West China Hospital, Sichuan University, Chengdu, Sichuan, China

*Corresponding author

Telephone numbers: 086-028-19983137868

Email: zhaoxudong@wchscu.cn

ORCID: 0000-0001-9126-7365

Additional file 1: Fig. S1

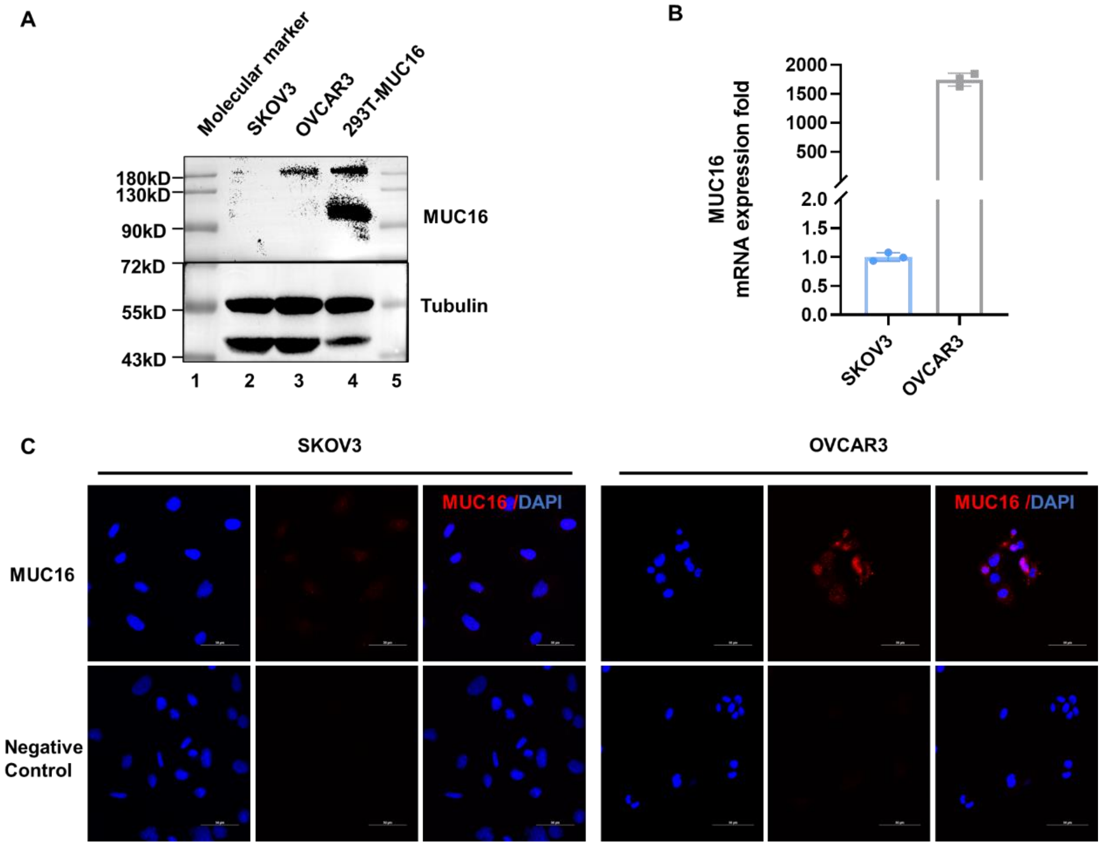

Additional file 1: Fig. S1 The expression of MUC16 in ovarian cells (SKOV3, OVCAR3). (A) In the main body of the text, the original Western blot image in Figure 1b was cropped to include all pertinent lanes and bands. **(B, C)** Quantification of RNA transcripts **(B)** and live cell immunofluorescence detection **(C)** for MUC16 in ovarian cells (SKOV3, OVCAR3). Representative confocal image of MUC16 protein expression in various ovarian cancer cell lines. Scale bars = 50 μm.
