# Supplementary material for: Mesothelin-based CAR-T cells exhibit potent antitumor activity against ovarian cancer
Source: J Transl Med. 2024 Apr 18;22:367. doi: 10.1186/s12967-024-05174-y (PMC11025286; doi:10.1186/s12967-024-05174-y)
Supplement: Supplementary file 2 — Additional file 2: Figure S2. Live cell immunofluorescence detectionof MUC16 overexpression in SKOV3 ovarian cells. [file 12967_2024_5174_MOESM2_ESM.pdf]

Additional file 2: Fig. S2

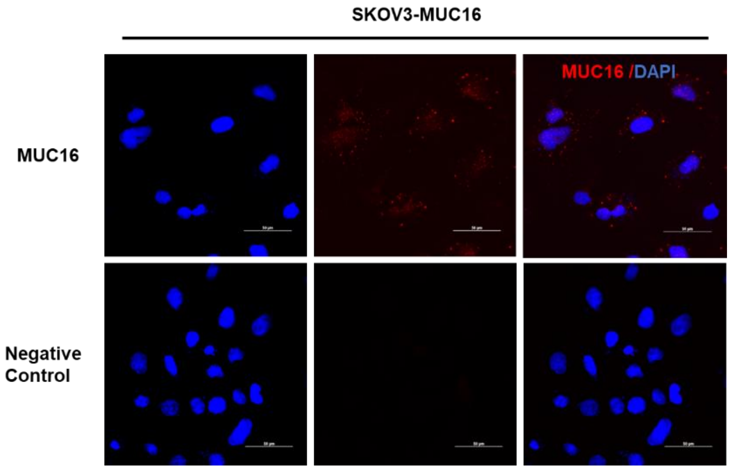

Additional file 2: Fig. S2 Live cell immunofluorescence detection of MUC16 overexpression in SKOV3 ovarian cells. Representative confocal image of MUC16 protein expression in SKOV3-MUC16 cell lines. Scale bars = 50 µm.
