# Supplementary material for: Mesothelin-based CAR-T cells exhibit potent antitumor activity against ovarian cancer
Source: J Transl Med. 2024 Apr 18;22:367. doi: 10.1186/s12967-024-05174-y (PMC11025286; doi:10.1186/s12967-024-05174-y)
Supplement: Supplementary file 3 — Additional file 3: Figure S3. Identification of ovariancancer stem-like cells. [file 12967_2024_5174_MOESM3_ESM.pdf]

Additional file 3: Fig. S3

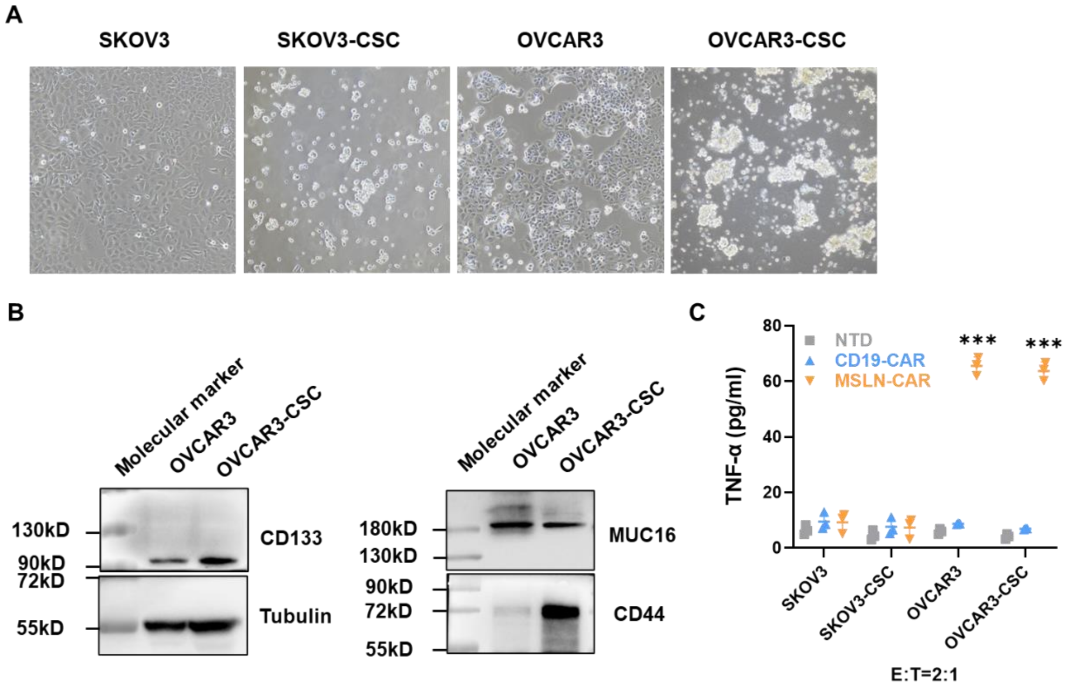

Additional file 3: Fig. S3 Identification of ovarian cancer stem-like cells. (A) Representative images of spheres formed by ovarian cancer stem cells. **(B)** Quantification of protein levels for CD133, CD44 and MUC16 in parental cells (OVCAR3) and cell line derived cancer stem cells (OVCAR3-CSC). Tubulin was used as a loading control. **(C)** ELISAs were used to detect TNF-α release by T cells in coculture supernatants. Three independent experiments were performed per condition. Statistics: two-tailed one-way ANOVA. Data are presented as the mean ± SD, $n = 3$; ***$P<0.001$ vs CD19-CAR or NTD.
