# Supplementary material for: Mesothelin-based CAR-T cells exhibit potent antitumor activity against ovarian cancer
Source: J Transl Med. 2024 Apr 18;22:367. doi: 10.1186/s12967-024-05174-y (PMC11025286; doi:10.1186/s12967-024-05174-y)
Supplement: Supplementary file 4 — Additional file 4: Figure S4. Detection of CD19-CAR and MSLN-CAR T cell typing and expression levels of depletion factors. [file 12967_2024_5174_MOESM4_ESM.pdf]

Additional file 4: Fig. S4

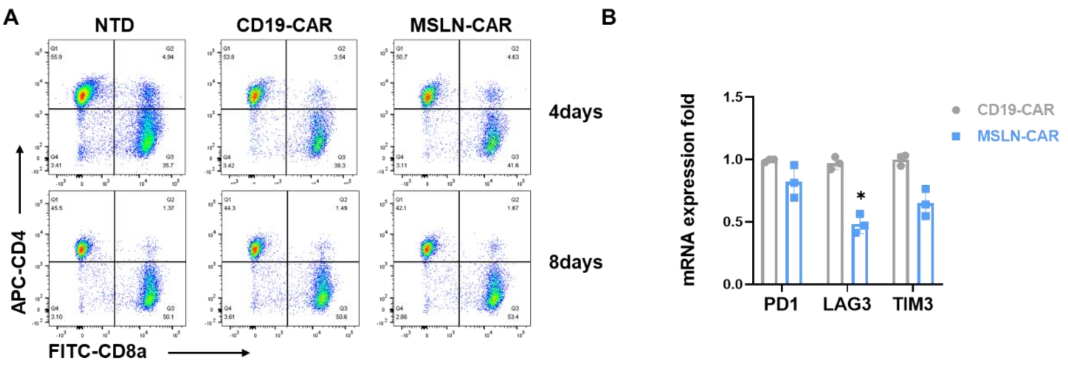

Additional file 4: Fig. S4 Detection of CD19-CAR and MSLN-CAR T cell typing and expression levels of depletion factors. (A) Flow cytometry was used to assess the percentages of $CD4^+$ and $CD8^+$ cells in CAR-T cultures on day 4 or 8 post-transduction (B) Statistical analysis of PD-1, LAG3 and TIM3 expression on CAR-T cells. CAR-T cells were incubated with OVCAR3 cells (E: T = 2: 1) for 3 days, either in culture medium. A and B consist of five biologic replicates and three technical replicates. For statistical analysis of two groups an unpaired two sample t -test is used. Data are presented as the mean ± SD, n = 3. *P<0.05 vs CD19-CAR.
