# Supplementary material for: Mesothelin-based CAR-T cells exhibit potent antitumor activity against ovarian cancer
Source: J Transl Med. 2024 Apr 18;22:367. doi: 10.1186/s12967-024-05174-y (PMC11025286; doi:10.1186/s12967-024-05174-y)
Supplement: Supplementary file 5 — Additional file 5: Figure S5. Infusion of MSLN-CAR T cells does not cause evident toxicity. [file 12967_2024_5174_MOESM5_ESM.pdf]

Additional file 5: Fig. S5

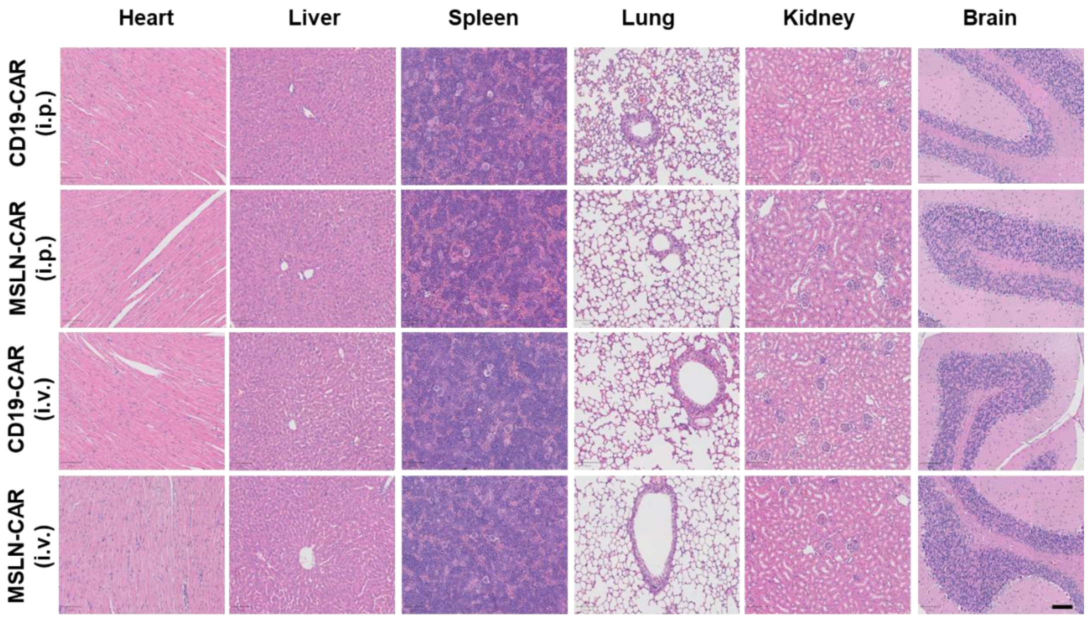

Additional file 5: Fig. S5 Infusion of MSLN-CAR T cells does not cause evident toxicity. NCG mice were intraperitoneal injected with $5x10^5$ OVCAR3 cells 5 days prior to receiving an infusion of T cells, and the major organs were harvested 5 days after the intravenous administration of T cells ($1x10^7$ cells/mouse). Pathological analysis of the indicated organs following hematoxylin and eosin staining on day 5 after the intraperitoneal or intravenous administration of CAR T cells. Micrographs are representative of the major organs from 3 mice per group. Scale bars = 100 μm.
