# Supplementary material for: Mesothelin-based CAR-T cells exhibit potent antitumor activity against ovarian cancer
Source: J Transl Med. 2024 Apr 18;22:367. doi: 10.1186/s12967-024-05174-y (PMC11025286; doi:10.1186/s12967-024-05174-y)
Supplement: Supplementary file 6 — Additional file 6: Table S1. STR Profile of Cell Lines. [file 12967_2024_5174_MOESM6_ESM.pdf]

Additional file 6: Table S1. STR Profile of Cell Lines

Cell Line	SKOV3	OVCAR3	HEK-293T	EAhy926
**D5S818**	11	11,12	8,9	10
**THO1**	9, 9.3	9, 9.3	7, 9.3	6, 9
**D13S317**	8,11	12	12	9, 12
**D16S539**	12	12	9,13	12
**vWA**	17,18	17	16,19	14, 16
**TPOX**	8,11	8	11	8
**D7S820**	13,14	10	11	12
**CSF1PO**	11	11	11,12	10, 11
**Amelogenin**	X	X	X	X
**Comparation with the databases from ATCC, DSMZ or JRCB**	No fingerprint match 21/10/26	No fingerprint match 21/10/26	No fingerprint match 21/11/18	No fingerprint match 21/11/18
