# Supplementary material for: Mesothelin-based CAR-T cells exhibit potent antitumor activity against ovarian cancer
Source: J Transl Med. 2024 Apr 18;22:367. doi: 10.1186/s12967-024-05174-y (PMC11025286; doi:10.1186/s12967-024-05174-y)
Supplement: Supplementary file 7 — Additional file 7: Table S2. The primer sequences. [file 12967_2024_5174_MOESM7_ESM.pdf]

Additional file 7: Table S2. The primer sequences

Genes	Organism	Forward primer (5'→3')	Reverse primer (5'→3')
**CD133**	Human	AGTCGGAAACTGGCAGATAGC	GGTAGTGTTGTACTGGGCCAAT
**CD44**	Human	CCAGAAGGAACAGTGGTTTGGC	ACTGTCCTCTGGGCTTGGTGTT
**CD117**	Human	CACCGAAGGAGGCACTTACACA	TGCCATTCACGAGCCTGTCGTA
**CD24**	Human	GAACAAAGCAAGGGCTTCGG	AGCCGGCGAGACCCTG
**PD1**	Human	AAGGCGCAGATCAAAGAGAGCC	CAACCACCAGGGTTTGGAACTG
**LAG3**	Human	GCAGTGTACTTCACAGAGCTGTC	AAGCCAAAGGCTCCAGTCACCA
**TIM3**	Human	AGACAGTGGGATCTACTGCTG	CCTGGTGGTAAGCATCCTTGG
**MUC16**	Human	CCAGTCCTACATCTTCGGTTGT	AGGGTAGTTCCTAGAGGGAGTT
**MSLN-CAR**	Human	TACGAGCAGCTGGACGTCCTAA	GGCCACGTCTCTTGTCCAAAAC
**18S rRNA**	Human	GTAACCCGTTGAACCCCATT	CCATCCAATCGGTAGTAGCG
