# Supplementary material for: Mesothelin-based CAR-T cells exhibit potent antitumor activity against ovarian cancer
Source: J Transl Med. 2024 Apr 18;22:367. doi: 10.1186/s12967-024-05174-y (PMC11025286; doi:10.1186/s12967-024-05174-y)
Supplement: Supplementary file 8 — Additional file 8: Table S3. Antibodies used in the study. [file 12967_2024_5174_MOESM8_ESM.pdf]

Additional file 8: Table S3. Antibodies used in the study

Antibody	Company	Art. No.	Apply (dilution)	References
**Tubulin**	Thermo Fisher Scientific	MA5-16308	WB (1:5000)	PMID: 29716620
**Mucin 16/MUC16**	Santa Cruz	sc-365002	WB (1:1000)<br>IHC (1:200)<br>FC (1:1000)	PMID: 32890404<br>PMID: 29716620<br>PMID: 29709661
**CD133**	Santa Cruz	sc-365537	WB (1:1000)	PMID: 37299411<br>PMID: 35359935
**CD44**	Proteintech	15675-1-AP	WB (1:2000)	PMID: 37291150
**CD3**	HUABIO	SY0239	IHC (1:200)	PMID: 37897831
**Anti-Rabbit IgG**	Sigma	A6154	WB (1:5000)<br>IHC (1:10000)	PMID: 24178031<br>PMID: 24820024
**Anti-Mouse IgG**	Sigma	A4416	WB (1:5000)<br>IHC (1:10000)	PMID: 32839551<br>PMID: 28679754
**Cy3 goat anti-Mouse IgG**	Invitrogen	A10521	ICC/IF (1:1000)	PMID: 37702564
**APC anti-human CD4**	Biolegend	357408	FC (1:1000)	PMID: 33563974
**APC/Cyanine7 anti-human CD8**	Biolegend	344714	FC (1:1000)	PMID: 33376221
**PE anti-human CD45RO**	Biolegend	304206	FC (1:1000)	PMID: 34673568
**APC anti-human CD197 (CCR7)**	Biolegend	353214	FC (1:1000)	PMID: 34673568
